# Perceptions of transmission of body weight and telemonitoring in patients with heart failure?

**DOI:** 10.3402/qhw.v8i0.21524

**Published:** 2013-12-16

**Authors:** Patrik Lyngå, Bengt Fridlund, Ann Langius-Eklöf, Katarina Bohm

**Affiliations:** 1 Department of Clinical Science and Education, Södersjukhuset, Karolinska Institutet, Stockholm, Sweden; 2 School of Health Sciences, Jönköping University, Jönköping, Sweden; 3 Department of Neurobiology, Care Sciences and Society (NVS), Karolinska Institutet, Huddinge, Sweden

**Keywords:** Body weight, heart failure, phenomenography, self-care, telemonitoring

## Abstract

Chronic heart failure (HF) is associated with a high burden of morbidity and mortality and with reduced quality of life. New techniques such as telemonitoring (TM) have recently been introduced in the care of patients with HF in order to improve outcomes. TM is defined as sending data from the patients’ home to healthcare professionals. Most studies have focussed on endpoints such as morbidity and mortality, and relatively little attention has been paid to patients’ perceptions of TM. Therefore, the aim of this study was to explore and describe patients’ perceptions of transmission of body weight (BW) and TM, regularly accomplished from patients’ homes to an HF clinic. An explorative design with a phenomenographic approach was used, and semi-structured interviews were conducted with a maximum variation sampling of 20 participants. The findings are described in five metaphoric categories that were assigned and used as a description: the habitual patient, the concerned patient, the technical patient, the secure patient, and the self-caring patient. The conclusions were that the transmission of BW made the patients active in their own care and increased self-care activities. In clinical care, concerns for deterioration in HF as well as the reliability of the TM system should be considered. Future research may focus on healthcare professionals and their perceptions of transmission of BW and TM in the care of patients with HF.

Patients with heart failure (HF) often report impaired quality of life, and increased self-care activities could have substantial importance for these patients (Hobbs et al., 2002). Non-compliance to self-care activities is also associated with an increased risk in morbidity and mortality (Moser et al., 2012; Van der Wal, van Veldhausen, Veeger, Rutten, & Jaarsma, 2010). However, it has been shown that knowledge in self-care does not automatically lead to improvement in self-care activities (Strömberg, Dahlström, & Fridlund, 2006). Despite education efforts to improve self-care, hospital readmission rates are still high, and the need for new methods is important to help improve care and cut healthcare costs (Cleland, Louis, Rigby, Janssens, & Balk, 2005). Recently, new techniques such as telemonitoring (TM), defined as transmission of data from the patient to healthcare professionals, have been developed (Inglis et al., 2008). Early feasibility studies have successfully tested the possibility of sending data such as blood pressure, body weight (BW), and electrocardiogram from the patient's home (De Lusignan, Wells, Johnson, Meredith, & Leatham, 2001; Jenkins & McSweneney, 2001; Nanewicz et al., 2000). TM in addition with symptom questions have been the focus in several randomized controlled trials (RCTs), and even though the results in meta-analyses showed significant decreases in hospitalization and death, the findings in large multicentre RCTs have been neutral, and thus, TM is mentioned as an alternative in HF care in the current guidelines (Chaudhry et al., 2010; Hunt et al., 2009; Inglis et al., 2008; McMurray et al., 2012). Another issue that raises important questions is the rather low compliance rate with TM that is reported in RCTs (Chaudhry et al., 2010; Cleland et al., 2005).

In developing new strategies for patient care, it is important to describe the patients’ perceptions of an innovative follow-up method such as TM in order to organize optimal care. Still, little attention has been paid to patients’ experiences of TM. Seto et al. (2012) described mainly positive experiences from patients and physicians using TM. Another qualitative study suggested that patients developed self-care skills in HF using TM (Riley, Gabe, & Cowie, 2013). Knowledge about patients’ experiences of a phenomenon and the variations within them has also been suggested to be an important tool when planning care for patients with chronic conditions (Larsson, Bergman, Fridlund, & Arvidsson, 2010). Of particular interest is to get a deeper understanding in how patients experience the care when it is moved outside of the hospital to their homes with the support of TM. This demands the patients to be more active in their own care (e.g, perform the transmission of BW) and, when necessary, receive feedback from their healthcare professionals (i.e., to increase the dose of medication). Previous studies have focussed on the effects of TM, but there is still a lack of knowledge of what it actually means to the patients. Therefore, the aim of this study was to explore and describe patients’ perceptions of transmission of BW and TM, regularly accomplished from patients’ homes to a HF clinic.

## Methods

An explorative and descriptive design with a phenomenographic approach was used (Marton, 1981). The study was a qualitative part of an RCT, involving six centres in Sweden initiated to reduce readmission rates in patients recently hospitalized for HF (Lyngå et al., 2012). Patients in the intervention group were given an electronic scale to place in their home and asked to weigh themselves daily. The weight measurements were automatically transmitted to an HF clinic. If a weight gain was detected of >2 kg in 3 days, the patients were contacted by telephone and asked questions based on the RCT study protocol. If there were signs of deterioration in the patients (e.g., increased breathlessness or tiredness, swollen legs or difficulties to lay flat in bed, together with increased BW), the dose of diuretics was temporally increased. The intervention group was compared with a control group in which patients were recommended daily weighing and, in case of weight gain >2 kg in 3 days, asked to contact the HF clinic for further measures, as recommended in the European Society of Cardiology (ESC) guidelines for HF (McMurray et al., 2012).

The goal of the phenomenographic approach is to describe how individuals in a group experience and understand a certain phenomenon in the world around them (Barnard, McCosker, & Gerber, 1999). This means studying people's ways of understanding a phenomenon and the variations within them (Marton, 1981). Phenomenography involves different ways of understanding, and both the “what” aspect, which tells us what the focus for the subject is, and the “how” aspect, which describe how meaning is created for him or her (Larsson & Holmström, 2007). In addition to the first-order perspective aiming to describe the world as it is, phenomenography also uses the second-order perspective (i.e., the variations in the understanding of the transmission of BW). The essence of phenomenography can be described as the variation in perceptions of a certain phenomenon and searching for the underlying structure of variance and how persons perceive the world in a cognitive way (Wenestam, 2000). Initially, phenomenography was used in educational research, but the approach has also shown to be useful in healthcare areas such as management and development of various healthcare services (Barnard et al., 1999).

## Participants

From the intervention group, who all had used the TM system in the RCT, 23 of 179 patients were asked to participate in the present study. Patients were sent written information about the study by mail and were then contacted by telephone 1–2 weeks later. The selection for the present study was done purposefully with a maximum variation sampling in order to ensure variation among the patients regarding sex (5 women, 15 men), civil status (5 living alone, 15 co-habiting), age (61–86 years, mean 74 years), education (7, < 9 years and > 9, 13 years), and study centres (Patton, 1990). Three patients declined participation, and 20 patients were finally included in the present study.

## Interviews

The interviews were conducted by the first author, but because he also worked with the RCT at one of the centres, the interviews at that particular centre were conducted by the last author. The interviews took place either in the patient's home or in a private room at the hospital. All interviews, which lasted between 13 and 45 min, were recorded and transcribed verbatim. Two pilot interviews were performed to test the semi-structured question guide, and as no revision of the questions was needed, the pilot interviews were included in the analyses. Before the interview started, the study aim was described to the participants (Kvale & Brinkmann, 2009). The interviews were held on a cognitive level according to the phenomenographic approach (Marton & Booth, 1997). The questions were as follows:

- You have participated in a study in which you have weighed yourself, and your weight has been transmitted to an HF clinic. What did the transmission of body weight mean to you?
- How was the transmission of body weight distinct to you in your self-care?
- Please, state any positive or negative aspects of transmitting body weight?

These questions were followed by probes such as “Could you tell more about that?” and “How: in what way?” in order to get as much information as possible from the interviewees. Finally, the interviewer summarized the conversation previously with the patient.

## Data analysis

The research team included all authors who together had a large clinical and methodological experience. Continuing discussions were held during the process. The interviews were analysed in seven steps in accordance to the phenomenographic approach outlined by Larsson and Holmström (2007).

1. The research team read the entire text several times individually.
2. Marks were made where patients gave answers to the three main interview questions corresponding to the aim of the study.
3. The marks were then compared and discussed to identify patients’ dominant way of understanding the transmission of BW (i.e., what was most important and in focus among the patients) and how it was described.
4. Descriptions based on similarities and differences were categorized.
5. The research team identified patients’ non-dominant ways of understanding of the phenomenon (i.e., other descriptions of understanding the phenomenon).
6. The research team created a structure of the descriptive categories in order to find a structure in the outcome space.
7. Five metaphoric categories were decided as a description of the findings.

It was necessary to constantly return to the various steps of the analysis until a final categorization was agreed on by the research team. This is in accordance to the negotiated consensus process of the phenomenographic tradition (Göransson, Dahlgren, & Lennerstrand, 1997).

## Ethics

Ethics approval was obtained from the Regional Ethics committee in Stockholm, Sweden (nr. 2005/537-31), and the study conforms to the principles outlined in the Declaration of Helsinki (2008). All patients gave verbal informed consent to participate in the study. Patients were also informed of their right to withdraw their consent to participate at any time and that confidentiality of information given in the interviews was guaranteed.

## Findings

From the 20 interviews, five metaphoric categories were identified. The dominant and non-dominant ways of understanding the phenomenon (i.e., the transmission of BW) are shown in Table I.

**Table I:** The different ways of understanding the transmission of body weight (BW) in a dominating (+ +) and non-dominating (+) way.

Patients m = male f = female	The habitual patient	The concerned patient	The technical patient	The secure patient	The self-caring patient
1 (m)		+	+	+ +	+
2 (m)	+		+ +	+	+
3 (m)	+	+ +	+		+
4 (m)		+		+ +	
5 (m)	+		+	+ +	
6 (f)	+		+	+ +	
7 (m)	+ +			+	+
8 (m)			+	+ +	+
9 (m)		+	+	+	+ +
10 (m)		+ +		+	
11 (f)	+	+ +		+	+
12 (m)	+		+ +	+	+
13 (m)	+			+	+ +
14 (f)	+ +			+	
15 (m)			+ +	+	+
16 (f)	+				+ +
17 (f)	+ +	+			
18 (m)	+ +		+	+	+
19 (m)	+				+ +
20 (m)	+ +		+		+

## The habitual patient

The metaphoric category “the habitual patient” contained perceptions that the transmission of BW and the daily weighing was easy to do and became a routine often done without reflecting on the phenomenon. The procedure was described as stressful because of concerns that they would forget to weigh themselves each morning; however, this was a temporary condition that transformed and turned into a routine. As one patient expressed it:

that turned into a daily morning routine. It [the scale] stood beside the bed, so it was just to step up on it and then: all done. There was no problem or difficulty or anything like that, not at all (No. 16).

For others, the transmission of BW became a ritual in the morning with a ceremonial character which was important in maintaining the self-care activity of daily weighing. As exemplified by this patient:

It became a ritual in the morning, to take the pills and weigh oneself …. Afterwards I can feel that when I don't have the scale, well, there is a piece of the ceremony missing … something about the seriousness has disappeared (No. 20).

The patients told that the weighing was easy to do and that it became a natural routine every morning, like brushing one's teeth or having breakfast.

## The concerned patient

The metaphoric category “the concerned patient” described concern in relation to the transmission of BW. One perception that was described was the fear to forget to carry out the daily activity of weighing oneself as well as assuming that they did something incorrect when something went wrong with the transmission of BW. This was described in the following ways: “it became like a small stress factor somehow” (No. 17); and “Then I thought, they must have switched it off but they had not …. Did I do something wrong? But, I had not done anything wrong” (No. 10).

There were also perceptions of pressure to do the weighing in the morning, which was described both as something good but also a form of coercion. One implication of the daily weighing was the fear of experiencing deteriorating HF condition, which caused concerns among patients. “I am a little frightened to step up on the scale. If I have gone up [in weight,] it will be fluid in the body and that can change rapidly” (No. 4).

## The technical patient

The descriptions from patients regarding technical matters led us to the metaphoric category “the technical patient.” The patients had different experiences of the equipment, the function of the electronic scale, and the modem. There were patients for whom the technical equipment was working very well, whereas for others, problems occasionally arose. One patient stated: “Yes, it was excellent. It can't be better. Because the only thing I did was to plug in the telephone in the wall, into the wall socket and push the start button” (No. 2). There were also indications that when the system did not work as expected, the patients’ enthusiasm decreased and they got weary of using the electronic scale. This was summarized by the following statement: “Sometimes it worked well and sometimes it did not. Well … then you got tired of it [the electronic scale]” (No. 15).

## The secure patient

Descriptions of being looked after and the sense of security that emerged led to the metaphoric category “the secure patient.” The patients had constant contact with the HF clinic and their healthcare professionals via the daily transmission of BW. This constant contact was perceived by the patients as receiving good care from the HF clinic. Being under the control of healthcare professionals in a chronic situation influenced the patients’ situation in a positive manner. As one patient stated:

Yes, they are probably keeping an eye on me. If something happens there is somebody on the other end that will see that; aha, something's happening here (No. 12).

There were also patients who wanted more contact with the HF clinic, wishing to be told that everything is fine and that there is no sign of deterioration. As one patient expressed it: “when everything was fine you did not get any feedback that: now you're doing really well …. I thought that was wrong” (No. 9).

## The self-caring patient

The patients considered that the transmission of BW was helpful in carrying out self-care activities. The metaphoric category “the self-caring patient” emerged based on patients’ descriptions of being active, wanting responsibility, and fully cooperating with the study nurses. There were patients who closely followed their weights, being aware when something was beginning to go wrong (e.g., an increase in BW). For one patient, the relation between increased BW and fluid retention became obvious.

A few times I guess, I noticed that I had put on too much [weight] and it was fluid. It was water or fluid in my body and the ankles became swollen (No. 13).

This was something that had not been reflected on before by this patient but now became significant: “I feel much better being in control of the situation which I was not before the study, if I may say so” (No. 13). Thus, the TM system acted as a doorway for patients to better understand their own bodies. There were also patients who were active in performing self-care on their own, making lists over their BW and expressed that they did not experience any benefits from the transmission of BW, as this patient said: “It [the transmission of BW] did not mean anything, because I kept control myself. I weigh myself and I write it down” (No. 19).

## Discussion

The main finding was that the patients perceived that their self-care was supported and encouraged by the transmission of BW through the TM system. This helped them keep abreast of their HF condition. The collaboration between patients and the HF clinic proved helpful to the patients who described that the TM made them understand the relation between increased BW and other HF symptoms such as swollen legs. Similar findings were reported by Riley et al. (2013) where patients with HF developed self-care skills and were empowered to make decisions about their own condition. It has been suggested that the cooperation between patients and specially trained nurses, within the concept of TM, is helpful in this process. It is also expressed by patients, in the present study, that being in control of the situation (i.e., having HF) was perceived positively. This finding is in line with a study of patients with rheumatism who wanted to be involved in the treatment and decision-making process related to their therapy (Larsson et al., 2010).

Patients in the present study also expressed that they could do the weighing themselves because it was easy to perform and that they did not need the transmission of BW. However, it might be argued that the measured compliance rates to the daily weighing were higher in the RCT compared to the self-reported levels in the European heart survey, suggesting that most patients do not perform daily weighing as a self-care activity to a desirable level (Lainscak et al., 2007; Lyngå et al., 2012). A perception of fear caused concerns among patients that the transmission of BW might remind the patients of illness and further deterioration in their health. This fear must be considered seriously as it has to do with their own understanding of the condition. It may also be so that weight gain involves an increase of the diuretic dose, which can cause inconvenience in the patient's daily life. Furthermore, the patients perceived that the transmission of BW caused considerable concern when the system did not work correctly, and patients thought it was their own fault even though this was not the case. Patients who experienced technical problems were less likely to use the electronic scales for the transmission of BW, indicating that a TM system needs to be reliable. In comparison to our study, a contrast was seen in a study of patients on palliative cancer treatment as the patients seemed to accept problems with the equipment when assessing pain with a digital pen and a pain diary (Lind, Karlsson, & Fridlund, 2008). One could speculate whether it was more crucial for patients who actually experience pain as compared to patients measuring and transmitting BW to determine whether a potential deterioration in HF is present. The patients also described the TM system as excellent and easy to use. Patients were more willing to do the daily weighing, although variation was noted. Thus, technology seemed helpful to increase this particular self-care activity. When discussing compliance rates in the RCT, other possibilities influencing the rates of daily weighing must be kept in mind, not just the technical function of the system but also the adherence to overall HF treatment (Lyngå et al., 2012).

The daily weighing was performed by routine and often without reflection. This is comparable to the routines of those patients who self-administered subcutaneous injections (Larsson et al., 2010). However, the daily electronic transmission of BW created a meaning for the patients and by that had some significance. This could be important because patients with HF are often more depressed than healthy individuals, and the feeling of participating might have a positive effect on these patients (Lesman-Leegte et al., 2009). Seto et al. (2012) described patient anxiety before their use of a TM system, but increased reassurance and reduced anxiety were reported after the patients began using the system. This was partly seen in the present study where patients initially experienced a temporary anxiety not to forget to do the daily weighing. The patients perceived that they were safe in the hands of the HF clinic. This was also noted by Seto et al. (2012) where patients indicated that the TM system was comparable to having a physician by their side all the time, which gave a strong sense of security. In addition, patients received a feedback message if everything was normal with the transmitted HF parameters, information that the patients identified as important. No such feedback was given in the main study, although patients noted that such feedback would have been desirable (Lyngå et al., 2012).

## Methodological considerations

Rigour in the data collection process and analysis was ensured by using the concepts of credibility, dependability, confirmability, and transferability (Polit & Beck, 2010). Because the patients’ perception of the phenomenon was the focus of this study, the phenomenographic approach supported a good credibility (Marton & Booth, 1997). Credibility was further strengthened by the use of the semi-structured interview guide which was developed to guarantee that all patients got the same questions. Patients were asked to reflect on their experience of the object of the study, follow-up questions were posed, and the patients were encouraged to talk openly. A characteristic of phenomenography is the search for variation, in which every perception that emerges is relevant and important (Marton & Booth, 1997). To increase credibility, each metaphoric category in this study was described by several patients. Dependability was strengthened by the fact that the data analysis sought to identify patients’ dominant and non-dominant ways of understanding the transmission of BW. To achieve confirmability, all steps of the analysis have been consciously reported, and consensus was achieved in the research team. The perceptions are described in detail in order to show that the chosen way of describing differences and similarities in the material is well supported, and quotations are used to show the relevance of the categories (Sjöström & Dahlgren, 2002). The variation in length of the interviews, which lasted between 13 and 45 minutes, can be questioned, as 13 min is unusually short for a phenomenographic interview. However, the informant gave interesting answers corresponding to the aim of the study and was therefore included in the analysis. Finally, transferability, the extent to which the findings can be transferred to other settings, was strengthened by the method and recruitment process. Thus, transferability to similar settings and patient groups is plausible.

## Conclusions

The transmission of BW was easy to perform, made patients with HF active in their own care, and increased their self-care activities. Patients perceived that they were safe and well cared for and got a better understanding of their own bodies. However, there were concerns of potential deterioration in HF related to weight gain which should be considered in clinical care.

## Clinical and research implications

An interactive approach in TM might be useful as the transmission of BW in the present study was not giving enough feedback to patients. TM systems have to be technically reliable and might be helpful as a complement to personal contacts between patients and HF clinics. In future studies, the interactive element of TM should be in focus and also include healthcare professionals’ perceptions of transmission of BW and TM in the care of patients with HF.

## References

[CIT0001] Barnard A, McCosker H, Gerber R (1999). Phenomenography: A qualitative research approach for exploring understanding in health care. Qualitative Health Research.

[CIT0002] Chaudhry S. I, Mattera J. A, Curtis J. P, Spertus J. P, Herrin J, Lin Z (2010). Telemonitoring in patients with heart failure. New England Journal of Medicine.

[CIT0003] Cleland J. G. F, Louis A. A, Rigby A. S, Janssens U, Balk A. H. M. M (2005). Non invasive home telemonitoring for patients with heart failure at high risk of recurrent admission and death. Journal of the American College of Cardiology.

[CIT0004] De Lusignan S, Wells S, Johnson P, Meredith K, Leatham E (2001). Compliance and effectiveness of a 1 year's home telemonitoring: The report of a pilot study of patients’ chronic heart failure. European Journal of Heart Failure.

[CIT0005] Göransson A, Dahlgren L. O, Lennerstrand G (1997). Changes in conceptions of meaning, effects and treatment of amblyopia: A phenomenographic analysis of interview data from parents of amblyopic children. Patient Education and Counseling.

[CIT0006] Hobbs F. D, Kenkre J. E, Roalfe A. K, Davies R. C, Hare R, Davies M. K (2002). Impact of heart failure and left ventricular systolic dysfunction on quality of life: A cross-sectional study comparing common chronic cardiac and medical disorders and a representative adult population. European Heart Journal.

[CIT0007] Hunt S. A, Abraham W. T, Chin M. H, Feldman A. M, Francis G. S, Ganiats T. G (2009). 2009 Focused update incorporated into the ACC/AHA 2005 guidelines for the diagnosis and management of heart failure in adults: A report of the American College of Cardiology foundation/American Heart Association task force on practice guidelines developed in collaboration with the International Society for Heart and Lung Transplantation. Journal of the American College of Cardiology.

[CIT0008] Inglis S. C, Clark R. A, McAlistair F. A, Ball J, Lewinter C, Cullington D (2008). Structured telephone support or telemonitoring programmes for patients with chronic heart failure. Cochrane Database Systematic Review.

[CIT0009] Jenkins R. L, McSweneney M (2001). Assessing elderly patients with congestive heart failure via in-home interactive telecommunication. Journal of Gerontological Nursing.

[CIT0010] Kvale S, Brinkmann S (2009). Interviews: Learning the craft of qualitative research interviewing.

[CIT0011] Lainscak M, Cleland J. G, Lenzen M. J, Nabb S, Keber I, Follath F (2007). Recall of lifestyle advice in patients recently hospitalized with heart failure: A EuroHeart failure survey analysis. European Journal of Heart Failure.

[CIT0012] Larsson I, Bergman S, Fridlund B, Arvidsson B (2010). Patients’ independence of a nurse for the administration of subcutaneous anti-TNF therapy: A phenomenographic study. International Journal of Qualitative Studies in Health and Well-being.

[CIT0013] Larsson J, Holmström I (2007). Phenomenographic or phenomenological analysis: Does it matter? Examples from a study on anaesthesiologists’ work. International Journal of Qualitative Studies on Health and Well-being.

[CIT0014] Lesman-Leegte I, Jaarsma T, Coyne J. C, Hillege H. L, Van Veldhuisen D. J, Sanderman R (2009). Quality of life and depressive symptoms in the elderly: A comparison between patients with heart failure and age- and gender-matched community controls. Journal of Cardiac Failure.

[CIT0015] Lind L, Karlsson D, Fridlund B (2008). Patients’ use of digital pen for assessment in advanced palliative home healthcare. International Journal of Medical Informatics.

[CIT0016] Lyngå P, Persson H, Hägg-Martinell A, Hagerman I, Hägglund E, Langius-Eklöf A (2012). Weight monitoring in patients with severe heart failure (WISH): A randomized controlled trial. European Journal of Heart Failure.

[CIT0017] Marton F (1981). Phenomenography: Describing conceptions of the world around us. Instructional Science.

[CIT0018] Marton F, Booth S (1997). Learning and awareness.

[CIT0019] McMurray J. J. V, Adamopoulus S, Anker S. D, Auricchio A, Böhm M, Dickstein K (2012). ESC Guidelines for the diagnosis and treatment of acute and chronic heart failure 2012. European Heart Journal.

[CIT0020] Moser D. K, Dickson V, Jaarsma T, Lee C, Strömberg A, Riegel B (2012). Role of self-care in the patient with heart failure. Current Cardiology Reports.

[CIT0021] Nanewicz T, Piette J, Zipkin D, Serlin M, Ennis S, De Marco T (2000). The feasibility of a telecommunication service in support of outpatient congestive heart failure care in a diverse patient population. Congestive Heart Failure.

[CIT0022] Patton M. Q (1990). Qualitative evaluation and research methods.

[CIT0023] Polit D. F, Beck C. T (2010). Essentials of nursing research: Appraising evidence for nursing practice.

[CIT0024] Riley J. P, Gabe J. P. N, Cowie M. R (2013). Does telemonitoring in heart failure empower patients for self-care? A qualitative study. Journal of Clinical Nursing.

[CIT0025] Seto E, Leonard K. J, Cafazzo J. A, Barnsley J, Masino C, Ross H. J (2012). Perceptions and experiences of heart failure patients and clinicians on the use of mobile phone-based telemonitoring. Journal of Medical Internet Research.

[CIT0026] Sjöström B, Dahlgren L. O (2002). Applying phenomenography in nursing research. Journal of Advanced Nursing.

[CIT0027] Strömberg A, Dahlström U, Fridlund B (2006). Computer-based education for patients with chronic heart failure: A randomized controlled multicentre trial of the effects on knowledge, compliance and quality of life. Patient Education and Counseling.

[CIT0028] Van der Wal M. H. L, van Veldhausen D. J, Veeger N. J. G. M, Rutten F. H, Jaarsma T (2010). Compliance with non-pharmacological recommendations and outcome in heart failure patients. European Heart Journal.

[CIT0029] Wenestam C-G, Fridlund B, Hildingh C (2000). The phenomenographic method in health research. Qualitative research methods in the science of health.

[CIT0030] World Medical Association Declaration of Helsinki (2008). Ethical principles for medical research involving human subjects. http://www.wma.net/e/policy/b3.htm.

